# Genetic differentiation and bottleneck effects in the malaria vectors *Anopheles farauti* and *Anopheles punctulatus* after an LLIN‐based vector control program in Papua New Guinea

**DOI:** 10.1002/ece3.10917

**Published:** 2024-02-15

**Authors:** John B. Keven, Rebecca Vinit, Michelle Katusele, Lisa J. Reimer, Peter A. Zimmerman, Stephan Karl, Edward D. Walker

**Affiliations:** ^1^ Department of Population Health and Disease Prevention, Program in Public Health University of California‐Irvine Irvine California USA; ^2^ Department of Entomology Michigan State University East Lansing Michigan USA; ^3^ Department of Microbiology and Molecular Genetics Michigan State University East Lansing Michigan USA; ^4^ Vector‐borne Diseases Unit Papua New Guinea Institute of Medical Research Madang Madang Province Papua New Guinea; ^5^ Department of Vector Biology Liverpool School of Tropical Medicine Liverpool UK; ^6^ Center for Global Health and Diseases, Pathology Department Case Western Reserve University Cleveland Ohio USA; ^7^ Australian Institute of Tropical Health and Medicine James Cook University Cairns Queensland Australia

**Keywords:** *Anopheles*, bottleneck, microsatellites, mosquito, population genetics

## Abstract

Implementation of long‐lasting insecticide‐treated net (LLIN) programs to control human malaria transmission leads to substantial reductions in the abundance of *Anopheles* mosquitoes, but the impact on the population genetic structure of the malaria vectors is poorly known, nor has it been investigated in Papua New Guinea, where malaria is highly endemic and where several species of *Anopheles* have vector roles. Here, we applied Wright's *F*‐statistic, analysis of molecular variance, Bayesian structure analysis, and discriminant analysis of principle components to microsatellite genotype data to analyze the population genetic structure of *Anopheles farauti* between and within the northern and southern lowland plains and of *Anopheles punctulatus* within the northern plain of Papua New Guinea after such a program. Bottleneck effects in the two malaria vectors were analyzed using Luikart and Cornuet's tests of heterozygosity. A large, panmictic population of *An. punctulatus* pre‐LLIN program diverged into two subregional populations corresponding to Madang and East Sepik provinces post‐LLIN distribution and experienced a genetic bottleneck during this process. By contrast, the *An. farauti* population existed as two regional populations isolated by mountain ranges pre‐LLIN, a genetic structure that persisted after the distribution of LLINs with no further geographic differentiation nor evidence of a genetic bottleneck. These findings show the differential response of populations of different vector species to interventions, which has implications for program sustainability and gene flow.

## Graphical abstract

In Papua New Guinea, a nationwide vector intervention program based on the use of long‐lasting insecticidal bed nets has been ongoing since 2009. While there are many reports of reduced vector abundance and malaria transmission rates attributed to the bed nets, the extent to which this outcome affected the population genetic structure and demographic parameters of the mosquito vectors in Papua New Guinea has not been investigated. This paper reports the findings of a study that addressed this knowledge gap.

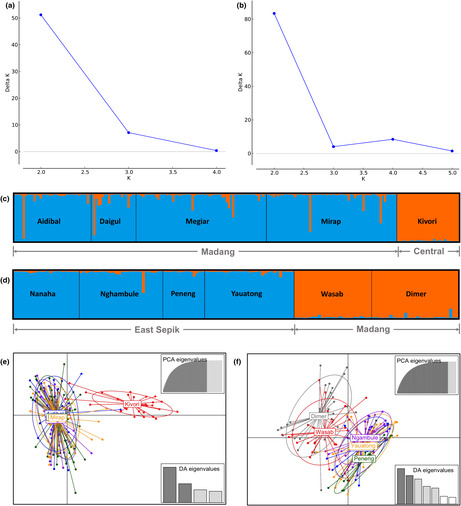

## 1 INTRODUCTION

Any event, whether natural or anthropogenic, that causes reduction in the abundance of a species can potentially reduce its effective population size and reshape its population genetic structure depending on the severity of the event. For example, the 72% reduction in the abundance of Peruvian fur seals (*Arctocephalus australis*) during the 1997–1998 El Nino Southern Oscillation resulted in a reduction in their effective population size, compromising their evolutionary potential to respond to future extreme events (Oliveira et al., 2009). A decline in the abundance of alpine caddisfly (*Allogamus uncatus*) during drought associated with the European open heat wave in the summer of 2003 reduced their effective population size and caused genetic differentiation in this species in the Swiss Alps (Shama et al., 2011).

In parts of the world where human malaria is endemic, control of the disease relies on the use of insecticides to target the *Anopheles* mosquitoes that transmit the parasites (WHO, 2011, 2015, 2019). As the intensity of parasite transmission depends on vector abundance, reduction of mosquito numbers by the insecticides commonly deployed as indoor residual spray (IRS) or long‐lasting insecticide‐treated nets (LLIN; WHO, 2011, 2015, 2019) results in reduced malaria transmission rates and ultimately infection prevalence in humans. In the past two decades, the implementation of IRS and LLIN against *Anopheles* mosquitoes has resulted in a considerable decline in the malaria burden globally (WHO, 2011, 2015, 2019). In many areas, the impact of IRS and/or LLIN on mosquitoes was severe, affecting the demographic parameters such as effective population size and changing the population genetic structure of the local *Anopheles* mosquitoes (Athrey et al., 2012; Ogola et al., 2019; Sougoufara et al., 2017; Wondji et al., 2005). For example, IRS campaign against malaria vectors on Bioko Island, Equatorial Guinea reduced the effective population size of *Anopheles gambiae* by 65%–92% compared to pre‐IRS estimates (Hodges et al., 2013). Another study in the same country found 55%–87% reduction in the effective population size of three other *Anopheles* species after IRS and LLIN campaign programs but this impact was not observed in areas where these programs were not implemented (Athrey et al., 2012). An LLIN program implemented in a Senegal village (Dielmo) in 2008 caused spatial structuration in the population genetics of *Anopheles arabiensis* (Sougoufara et al., 2017). These studies show that mosquito populations undergo demographic change in concert with the implementation of vector control methods. The impact of these vector control methods on effective population size has important implications for the evolutionary potential and fitness of the vectors, and the desired outcome is a negative impact or reduction in these two ecological quantities. A vector population with low genetic diversity due to bottleneck effect has a lower fitness and potential to adapt to environmental change such as new habitat types, pathogen strains, or temperature level which may indirectly affect their vectorial capacity. Knowledge about their population structure and gene flow is useful, for example, in monitoring and mitigating the spread of undesirable genetic traits such as insecticide resistance alleles among populations. It is also useful for guiding the implementation of novel vector control methods such as gene drive systems to propagate desired traits such as malaria parasite refractory genes into vector populations (Huang et al., 2011). The impact of these vector control methods on the demographic parameters and population genetic structure of mosquito vectors can be evaluated by analyzing genetic data such as DNA sequence haplotypes, microsatellite markers, and single nucleotide polymorphisms.

Human malaria is endemic in Papua New Guinea (PNG). The parasites of four species of malaria are transmitted in PNG by several species of *Anopheles* mosquitoes, particularly members of the *Anopheles punctulatus* group. Of the 13 known mosquito species that comprise this group (Beebe et al., 2015), *Anopheles koliensis*, *Anopheles farauti* sensu stricto and *An. punctulatus* sensu stricto are the primary vectors, owing to their widespread distribution and abundance throughout PNG (Burkot et al., 1988, 1989, 1990; Hetzel et al., 2016; Hii et al., 2001; Reimer et al., 2016). This study focused only on *An. farauti* and *An. punctulatus*; samples of *An. koliensis* were unavailable for inclusion. To control malaria in PNG, a nationwide LLIN program has been ongoing since 2009 (Hetzel, 2009; Hetzel et al., 2012; Hetzel, Choudhury, et al., 2014; Hetzel, Pulford, et al., 2014). A decline in malaria infection prevalence and transmission intensity was observed immediately after the LLIN program was implemented in 2009 (Hetzel et al., 2015, 2016, 2017; Koepfli et al., 2017; Reimer et al., 2016). This effect was attributed to a three‐fold reduction in *Anopheles* abundance measured as the vector landing rate on humans, observed at five sites in PNG where measure was 81 landings per person‐night before the LLIN program and 31 landings per person‐night after the program (Hetzel et al., 2016). In an inland area of East Sepik province where *An. punctulatus* is the dominant vector, the landing rates of this species declined by ca. six fold from a range of 6.4–61.3 bites per person‐night among villages before the LLIN program to 1.1–9.4 bites per person‐night after the program (Reimer et al., 2013). A similar trend was observed in villages along the north coast of Madang province where both vector species cohabit (Reimer et al., 2016). These results show that the abundance of malaria vectors in PNG was affected markedly after the LLIN campaign was implemented. However, the extent to which this outcome affected the population genetic structure and demographic parameters of the mosquito vectors has not been investigated.

The objective of this study was to test the hypothesis that the implementation of LLINs reduced abundance of *An. farauti* and *An. punctulatus* in PNG such that effective population size also declined and large, undifferentiated populations fragmented into smaller, differentiated ones.

## 2 MATERIALS AND METHODS

### 2.1 Study sites and mosquito samples

**FIGURE 1:**
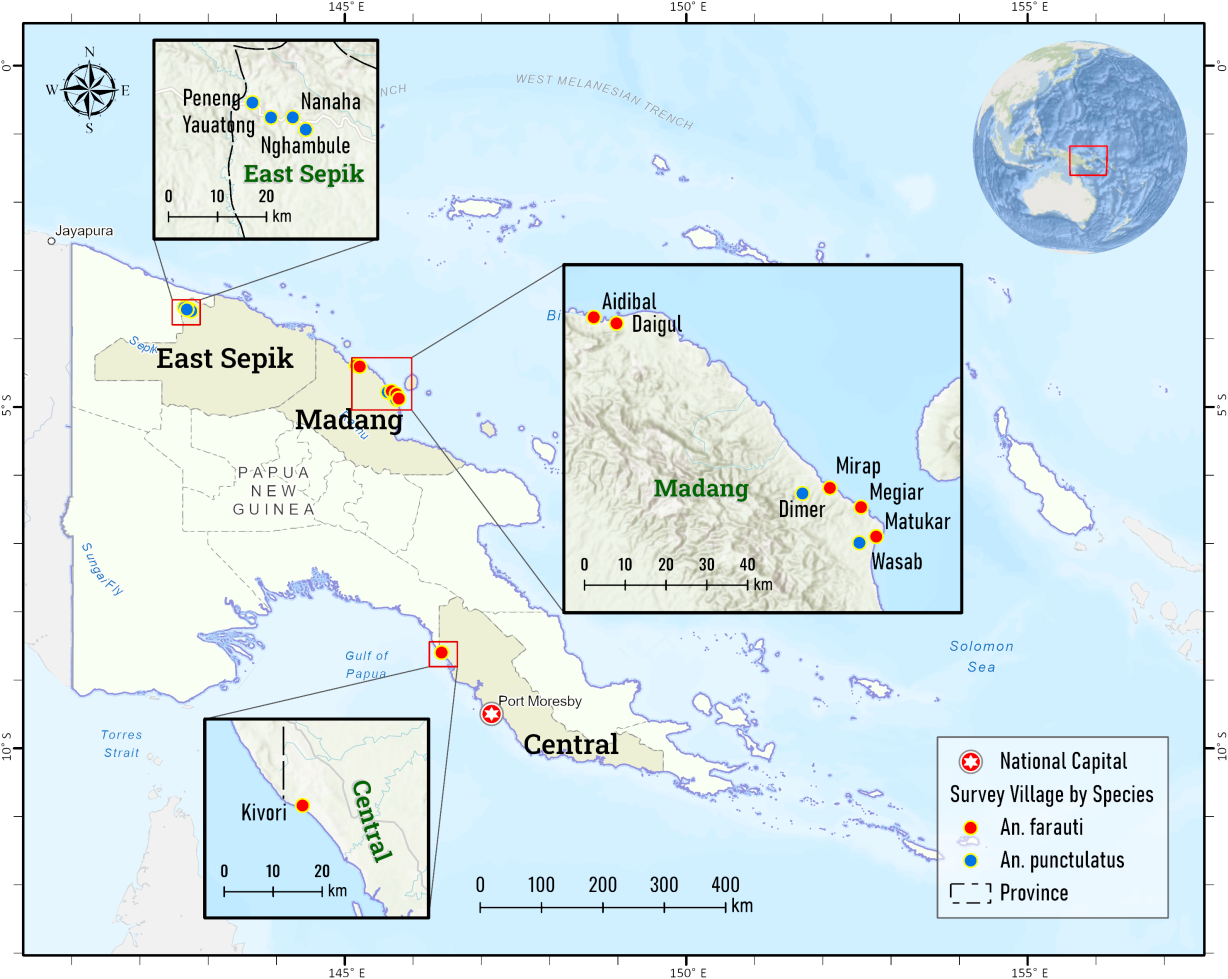
Sampling sites (villages) of *Anopheles farauti* (red dots) and *Anopheles punctulatus* (blue dots) in East Sepik, Madang, and Central provinces, Papua New Guinea.

The mainland of PNG is divided into northern and southern lowland plains by massive, connected chain of mountain ranges originating from the middle of the PNG‐Irian Jaya (a province of Indonesia) border in the west and extends to the southeasternmost tip of the mainland (Figure S1). Adult female mosquitoes were collected by barrier screen sampling or human landing catch methods as part of malaria transmission and vector ecology studies (Keven et al., 2017; Keven, Katusele, et al., 2019; Reimer et al., 2016) from 12 villages in three provinces in PNG (Figure 1). Of the three provinces, two (Madang and East Sepik) are located within the northern plain whereas the other (Central) is located in the southern plain (Figure 1). In this paper, a “sample” is defined as mosquitoes that were collected in a particular locality and year and is represented by a row in Table 1. Each locality is actually a village of no more than 1 km in radius. Thus, mosquitoes collected from the same locality but in different years are considered as different samples. Samples of *An. farauti* originated from six villages. These are Aidibal and Daigul in Bogia district, Madang province; Matukar, Megiar, and Mirap in Sumkar district, Madang province; and Kivori, Central province. The *An. farauti* samples represented a single year of collection (2017) in Kivori, Aidibal, Daigul, and Megiar, 2 years of collections (2010 and 2012) in Matukar, and 3 years of collections (2010, 2012, and 2017) in Mirap (Table 1). Samples of *An. punctulatus* originated from six villages which are different from those where *An. farauti* were collected. These are Dimer and Wasab in Sumkar district, Madang province, and Nanaha, Nghambule, Peneng, and Yauatong in Drekikier district, East Sepik province. The *An. punctulatus* samples represented a single year of collection (2012) in the two villages in Madang and 2 years of collections (2008 and 2010) in the four villages in East Sepik (Table 1). For clarity and consistency hereafter, the word “regions” refer to the northern and southern lowland plains of PNG, subregions refer to the provinces within a region, and localities refer to villages within a subregion where a sample of mosquitoes originates.

**TABLE 1:** Samples (represented by rows) of mosquitoes of the two vector species along with information about the province, district, village and year of collection, and the number of mosquitoes (*N*) in each sample.

Vector	Province	District	Village	Year	*N*
*Anopheles farauti*	Madang	Sumkar	Matukar	2010	47
		Sumkar	Matukar	2012	30
		Sumkar	Mirap	2010	27
		Sumkar	Mirap	2012	35
		Sumkar	Mirap	2017	61
		Sumkar	Megiar	2017	61
		Bogia	Aidibal	2017	36
		Bogia	Daigul	2017	21
	Central	Kivori	Kivori	2017	29
*Anopheles punctulatus*	Madang	Sumkar	Dimer	2012	29
		Sumkar	Wasab	2012	26
	East Sepik	Drekikier	Nanaha	2008	31
		Drekikier	Nanaha	2010	22
		Drekikier	Nghambule	2008	29
		Drekikier	Nghambule	2010	28
		Drekikier	Peneng	2008	32
		Drekikier	Peneng	2010	14
		Drekikier	Yauatong	2008	28
		Drekikier	Yauatong	2010	30

### 2.2 Microsatellite markers and primer design

The genomes of both species have been sequenced and are available in the National Center for Biotechnology Information (NCBI) Sequence Read Archive with accession number SRX555047 and SRX555048 for *An. punctulatus* and *An. farauti*, respectively (Logue et al., 2015). The genomes were screened for tetranucleotide or trinucleotide microsatellite repeats. Regions of the genome containing tandem repeats of a tetranucleotide motif (e.g., TGGA) or a trinucleotide motif (e.g., GAT) were detected by aligning a query sequence containing 50 repeats of the nucleotide motif with the genome sequence using Basic Local Alignment Search Tool (BLAST). From the BLAST outputs, regions of the genome that contained eight or more perfect (uninterrupted) repeats of the nucleotide motif were selected and primers were developed to anneal to the flanking regions of the microsatellite sequences. Primer design and quality testing were performed following the same method that was used for pig microsatellites described elsewhere (Keven, Walker, et al., 2019). Briefly, both the forward and reverse primers were designed with AA at the 3′ end to help reduce the formation of primer dimers during PCR (Innis & Gelfand, 1999). The specificity of each primer pair was evaluated by in silico tests of non‐specific binding against a wide range of nucleotide sequences in the NCBI database using Primer‐BLAST tool (Ye et al., 2012). The 5′ end of each forward primer was modified by adding a tag sequence complementary to one of four fluorescent‐labeled universal primers (Table S1). To promote complete adenylation of PCR amplicons, the 5′ end of the reverse primer was also modified by adding the “pig tail” sequence GTTTCTT (Brownstein et al., 1996). The procedure described above yielded 10 tetranucleotide microsatellite markers for *An. punctulatus* and seven markers (six tetranucleotide and one trinucleotide) for *An. farauti*. To achieve a total of 10 markers for *An. farauti*, three trinucleotide markers from a published source were adopted (Ambrose et al., 2014). The adopted primers were also modified at the 5′ and 3′ ends to be consistent with those developed in the current study. The microsatellite markers, their repeat motif, primer sequences, and allele range are presented for *An. farauti* in Table S1 and *An. punctulatus* in Table S2. Of the 10 *An. farauti* forward primers, three were tagged with the sequence complementary to universal primer 1 which was labeled with the fluorophore 6‐FAM, three were tagged with the sequence complementary to universal primer 2 labeled with PET, two were tagged with the sequence complementary to universal primer 3 labeled with NED and two were tagged with the sequence complementary to universal primer 4 labeled with VIC (Table S1). The same approach was taken on the forward primers of *An. punctulatus* (Table S2).

### 2.3 PCR amplification and genotyping

DNA was extracted from each mosquito using the DNeasy Blood and Tissue Kit (Cat. No. 69506, Qiagen, Valencia, CA). Species identification was confirmed using polymerase chain reaction (PCR) restriction fragment length polymorphism (Beebe & Saul, 1995). DNA sample of each mosquito was analyzed in a multiplex PCR that co‐amplified all 10 microsatellite loci. The PCR reaction was performed following the same method described elsewhere (Keven, Walker, et al., 2019). PCR products were analyzed by capillary electrophoresis (ABI 3730 Genetic Analyzer, Applied Biosystems, Foster City, CA) with LIZ 500 (Applied Biosystems) as internal size standard. Genotypes were determined using Peak Scanner software version 1.0 (Applied Biosystems) and alleles were represented by fragment size in base pairs. True off‐ladder alleles were distinguished from false off‐ladder calls due to rounding errors in allele scoring (based on the local Southern algorithm) by overlaying the electropherogram of all samples and manually identifying those samples with true off‐ladder peaks.

### 2.4 Data analyses

#### 2.4.1 Quality assessments

The microsatellite genotype data for each species were encoded in Genepop file format and are presented in Data S1 for *An. farauti* and Data S2 for *An. punctulatus*. The quality of the microsatellite data can be affected by the presence of null alleles, which are alleles that failed to amplify in the PCR due to mutations in the primer‐binding sites that prevent the primers from annealing properly (Brookfield, 1996). This can affect the data by biasing allele and genotype frequencies. However, the effect of null alleles on the estimates of population genetic parameters is negligible when the mean frequency of null alleles (averaged across loci) of a sample is ≤0.08 (Oddou‐Muratorio et al., 2009). In this study, the frequency of null alleles for each locus was estimated using the methods of Chapuis and Estoup (2007) in the software FreeNA.

The quality of a panel of microsatellite markers for use in population genetic analyses also depends on the independence of the markers. That is to say that two microsatellite markers are not independent of each other if they are genetically linked and are inherited together. Independence of pairs of microsatellite loci (null hypothesis) was tested through linkage disequilibrium (LD) analysis. The LD analysis was performed in the software Genepop 4.7 (Raymond & Rousset, 1995; Rousset, 2008) with the parameter Markov chain dememorization set to 1000, number of batches set to 500, and number of iterations set to 1000. Independence of a pair of loci was determined based on Bonferroni‐corrected *p* value, calculated as *p* = .05/(L*N), where *L* is the number of microsatellite loci and *N* is the number of mosquito samples. The data were also analyzed for Hardy–Weinberg equilibrium (HWE) using the exact test (Guo & Thompson, 1992) in Genepop with the same parameter setting described for LD tests. Bonferroni‐corrected *p* value, calculated as *p* = .05/*L*, was used to determine if a sample of mosquitoes was from a population that was in HWE at each microsatellite locus.

#### 2.4.2 Genetic differentiation

Genetic differentiation between regions, within regions, and among localities within subregions was tested using analysis of molecular variance (AMOVA; Excoffier et al., 1992). In addition to AMOVA, genetic differentiation was quantified using pairwise *F*_*ST*_ (Wright's *F*‐statistic; Wright, 1965). Unlike AMOVA which quantifies genetic differentiation by estimating Euclidean distances between alleles, Wright's *F*‐statistic quantifies it based on allele frequencies. Pairwise *F*_*ST*_ was calculated between pairs of samples and the resulting values were then used to quantify gene flow based on Wright's formula *N*_*m*_ = (1‐*F*_*ST*_)/4*F*_*ST*_, where *N*_*m*_ is the number of migrants per generation between two populations (Wright, 1931). The level of genetic differentiation between two regions or subregions was quantified by calculating the pairwise *F*_*ST*_ and *N*_*m*_ of all pairs of samples between them and then taking the mean value of each quantity. Similarly, the level of genetic differentiation within each region or subregion was quantified by calculating the pairwise *F*_*ST*_ and *N*_*m*_ of all pairs of samples within them and then taking the mean value of each quantity. The mean of pairwise *F*_*ST*_ or *N*_*m*_ values of sample pairs within subregions was compared to the mean values of sample pairs between subregions using Student's *t*‐test to determine variation in the level of genetic differentiation and gene flow within (local) and between subregions (regional). For *An. punctulatus* in East Sepik where samples were collected before and after the LLIN campaign, mean pairwise *F*_*ST*_ and *N*_*m*_ were also calculated for samples collected before and after the event and compared using *t*‐test. Analyses of AMOVA, *F*_*ST*_, and *N*_*m*_ were performed in the Microsoft Excel program GenAlex (Peakall & Smouse, 2006, 2012).

#### 2.4.3 Cluster analysis

Analysis of genetic clusters was performed using the model‐based Bayesian clustering method implemented in the software Structure (version 2.3; Pritchard et al., 2000). This method utilizes admixed population model to partition multilocus genotype data into a number of genetic clusters *K* and then calculates the likelihood of each *K* (Pritchard et al., 2000). The likelihood of *K* is the probability that the mosquitoes from which the genotype data were obtained come from *K* admixed populations. In Structure, the likelihood of each *K* (ranging from one to five or six) was simulated by setting the length of burn‐in period to 10,000, number of Markov chain Monte Carlo replicates after burn‐in to 10,000, and number of iterations to 10. The output of Structure analysis was analyzed in the web‐based programs Structure Harvester (Earl & vonHoldt, 2012) to generate plots of Δ*K* and Clumpak (Kopelman et al., 2015) to generate Bayesian cluster plots. Values of *K* that best explain the mosquito data were determined using the Δ*K* method of Evanno et al. (2005) implemented in Structure Harvester. In addition to the Bayesian method, genetic clustering was also analyzed using discriminant analysis of principal components (DAPC) method (Jombart et al., 2010) performed in R software (version 3.4.2) using functions available in the package *Adegenet* (Jombart, 2008). Unlike the Bayesian method, DAPC is a non‐model‐based multivariate data analysis. DAPC involves performing principal component analysis on the multilocus genotype data followed by the application of discriminant analysis with *K*‐means clustering approach (since the number of populations was unknown a priori) on the principal components to determine the number of clusters represented by the data (Jombart et al., 2010).

#### 2.4.4 Genetic diversity

Genetic diversity is a property of a biological population. Therefore, samples from various spatial locations within a population must be combined as a single, large sample and analyzed for genetic diversity. For each of the two malaria vector species, mosquitoes from localities that comprise the same genetic cluster determined by the AMOVA, Bayesian and DAPC analyses were combined as a single population, stratified (grouped) by year of collection and analyzed for number of alleles (*N*_*A*_), allelic richness (*A*_*R*_), observed heterozygosity (*H*_*O*_), expected heterozygosity (*H*_*E*_), and unbiased expected heterozygosity (*uH*_*E*_). The quantities *N*_*A*_, *H*_*O*_, *H*_*E*_, and *uH*_*E*_ were estimated using GenAlex (Peakall & Smouse, 2006, 2012) whereas *A*_*R*_ was estimated using *allel.rich* function of the R package *PopGenReport* (Adamack & Gruber, 2014).

#### 2.4.5 Tests of genetic bottleneck

As with the analysis of genetic diversity, for each population of *An. farauti* or *An. punctulatus*, mosquitoes were grouped by year of collection and genetic bottleneck was tested on each group using Luikart and Cornuet's tests of heterozygosity (LCH; Cornuet & Luikart, 1996). This test is based on the principle that a population that had undergone a reduction in effective population size experiences a reduction in both *N*_*A*_ and *H*_*E*_ at polymorphic loci, but *N*_*A*_ reduces faster than *H*_*E*_. Such a population would tend to exhibit higher *H*_*E*_, which is calculated from allele frequencies, than heterozygosity expected under mutation‐drift equilibrium (*H*_*EQ*_), which is calculated from *N*_*A*_ (Cornuet & Luikart, 1996). *H*_*E*_ and *H*_*EQ*_ were estimated for each microsatellite locus and Wilcoxon sign‐rank test was used to compare the median values (calculated across loci) of the two quantities. Quantification of *H*_*EQ*_ was performed assuming that the microsatellite loci mutate following the infinite allele mutation model (IAM; Kimura & Crow, 1964). IAM was chosen instead of two‐phase or stepwise mutation models because LCH test has been demonstrated only for markers that evolve under this mutation model (Maruyama & Fuerst, 1985). Populations with *H*_*E*_ > *H*_*EQ*_ were considered to have undergone a recent bottleneck event (Cornuet & Luikart, 1996). This analysis was performed using the software Bottleneck version 1.2.02 (Piry et al., 1999). It is important to note that unlike other tests which involve a comparison of two samples collected before and after an event such as the LLINs to make an inference about its effect on the population genetics of an organism, LCH makes an inference based only on a single sample collected after the event (Cornuet & Luikart, 1996). As most populations of the two mosquitoes in this study lacked samples collected before the LLIN campaign, we used LCH test to determine genetic bottleneck.

## 3 RESULTS

### 3.1 Data quality

In this study, the effect of null alleles on the estimates of population genetic parameters is negligible because the mean frequency of null alleles in all samples of both mosquito species was ≤0.08 except for the 2010 sample of *An. punctulatus* from Yauatong where the mean frequency was 0.09 (Tables S3 and S4). However, this value was not significantly greater than 0.08 based on one‐sample *t*‐test (*t* = 0.24, df = 9, *p* = .41). The results of LD and HWE are presented in Figures S2 and S3, respectively. For *An. farauti*, 10 loci and nine samples resulted in 405 pairwise LD tests. Of these, only two tests (TRI‐5 vs. AF1‐4 and TRI‐1 vs. AF1‐9) were found to be in LD; the rest were unlinked (Figure S2A). Similarly, of 450 pairwise tests for *An. punctulatus* (10 loci and 10 samples), only two were found to be in LD; the rest were unlinked (Figure S2B). As all four LD cases were found in only one mosquito sample, rather than all or several of the samples, it is likely that these cases were the result of population‐specific factors and not true genetic linkage. The results of HWE tests for *An. farauti* show that only three of 90 tests (10 loci by nine samples) deviated from HWE (Figure S3A). For *An. punctulatus*, 12 of 100 tests (10 loci by 10 samples) deviated from HWE (Figure S3B). These deviations from HWE might be caused by random genetic drift or sampling error.

### 3.2 Genetic differentiation

AMOVA was performed on three groups of mosquito data separately. These were *An. farauti* collected from five localities in Madang and Central provinces in 2017, *An. punctulatus* collected from six localities in Madang and East Sepik provinces in 2010 or 2012 and *An. punctulatus* collected from four localities in East Sepik province in 2008. The results of these three AMOVA analyses are presented in Table 2. The results show that for *An. punctulatus* collected in 2008 in East Sepik when LLINs were not yet distributed, there was no evidence of genetic differentiation among the localities. For the *An. punctulatus* samples collected after the LLIN campaign in 2010 or 2012 in Madang and East Sepik, genetic differentiation between the two subregions was significant and accounted for 4% of the total genetic variation. There was also significant differentiation among localities within subregions and accounted for 1% of the total genetic variation. For *An. farauti*, genetic differentiation between regions was significant and accounted for 11% of the total genetic variation. However, there was no genetic differentiation within regions.

**TABLE 2:** Results of hierarchical AMOVA analysis showing levels of genetic structure for (i) *Anopheles punctulatus* collected in four localities in a subregion (East Sepik) in 2008 (pre‐LLIN), (ii) *An. punctulatus* collected in six localities in two subregions (Madang and East Sepik) in 2010 or 2012 (post‐LLIN), and (iii) *Anopheles farauti* collected in five localities in two regions (northern and southern plains) in 2017 (post‐LLIN).

Source of variation	df	SS	MS	Var	%	*F*‐statistic	*p* value
Analysis 1. *An. punctulatus* (pre‐LLIN)							
Among samples	3	8.4	2.79	0.00	0	−0.004	.982
Among individuals	116	426.6	3.68	0.38	12	0.115	.001*
Within individuals	120	350.0	2.92	2.92	88	0.111	.001*
Total	239	785.0		3.30	100		
Analysis 2. *An. punctulatus* (post‐LLIN)							
Among provinces (East Sepik/Madang)	1	27.2	27.18	0.16	4	0.045	.001*
Among samples	4	19.3	4.83	0.02	1	0.006	.018*
Among individuals	143	550.8	3.85	0.46	13	0.051	.001*
Within individuals	149	436.0	2.93	2.93	82	0.137	.001*
Total	297	1033.3		3.57	100		
Analysis 3: *An. farauti* (only post‐LLIN)							
Among regions (South/North plains)	1	35.4	35.41	0.33	11	0.108	.001*
Among samples	3	6.5	2.18	0.00	0	−0.003	.963
Among individuals	203	591.2	2.91	0.18	6	0.105	.001*
Within individuals	208	532.0	2.56	2.56	83	0.065	.001*
Total	415	1165.1		3.07	100		

*Note*: Estimation of *p* value was based on 999 permutations across the full dataset. Asterisk (*) indicates significant *p* values.

Abbreviations: %, percentage of total variance; df, degrees of freedom; MS, mean sum of squares; SS, sum of squares; Var, variance components.

**FIGURE 2:**
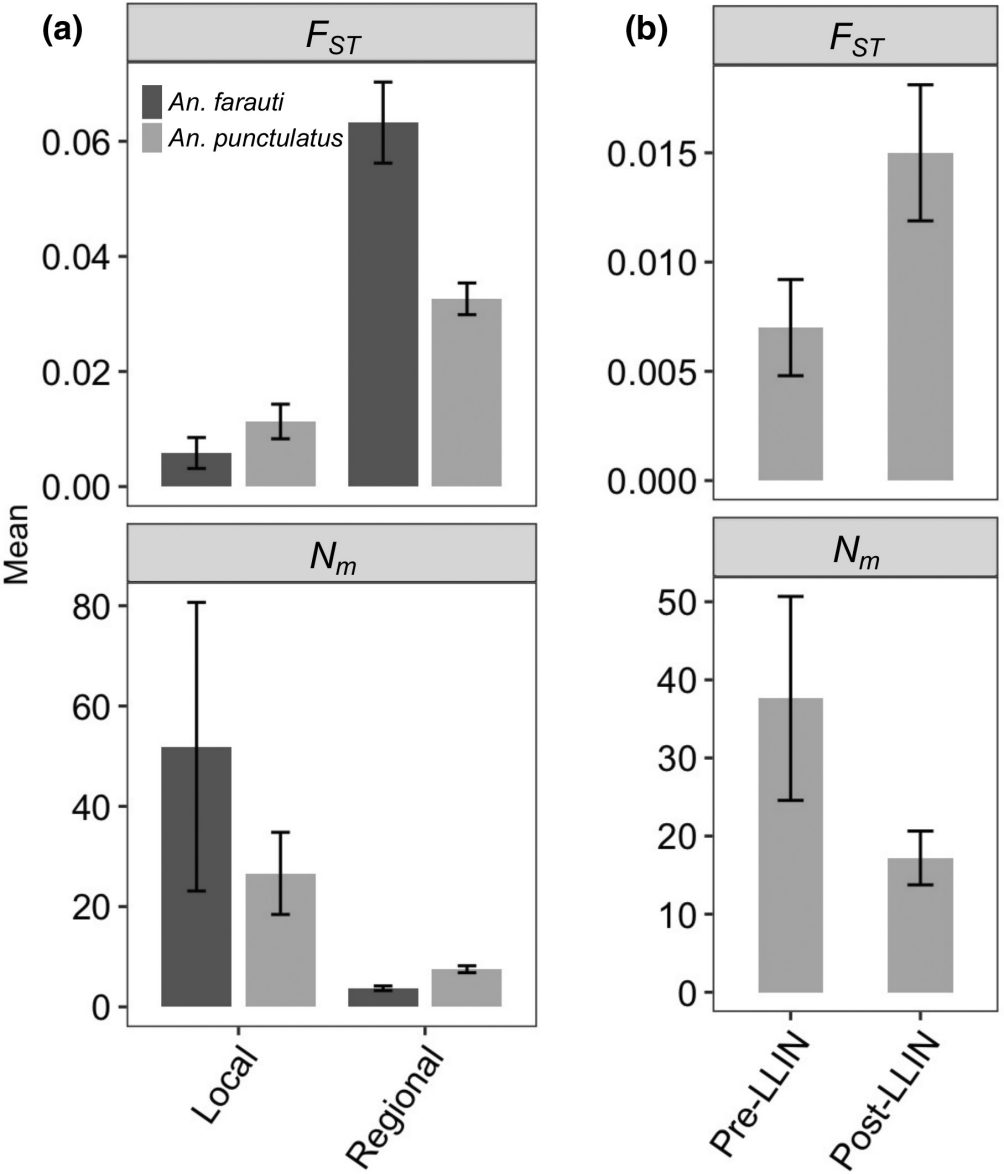
Mean pairwise *F*_*ST*_ and *N*_*m*_ averaged across sample pairs. (a) Comparison of mean *F*_*ST*_ and *N*_*m*_ for pairs of mosquito samples within province (local) and between provinces (regional). Results for *Anopheles farauti* were based on pairs of samples collected in 2017 and for *Anopheles punctulatus* were based on pairs of samples collected in 2010 or 2012. (b) Comparison of mean *F*_*ST*_ and *N*_*m*_ for *An. punctulatus* samples collected in East Sepik before (2008) and after (2010) implementation of the LLIN program. Error bars are 95% confidence interval of the means.

Pairwise *F*_*ST*_ and *N*_*m*_ were calculated for all samples in each of the three groups of mosquitoes described above and the results for both quantities were categorized into local and regional (including subregional) pairs as well as into pre‐LLIN and post‐LLIN pairs (Table S5). For *An. farauti*, the mean pairwise *F*_*ST*_ for local pairs (averaged across all sample pairs) was significantly lower than regional pairs (Figure 2a; *t* = −23.5, df = 4, *p* < .001). Correspondingly, the mean *N*_*m*_ for local pairs was significantly higher than regional pairs (Figure 2a; *t* = 4.3, df = 5, *p* = .008). Similarly, for *An. punctulatus*, the mean pairwise *F*_*ST*_ was significantly lower for local than regional pairs (Figure 2a; *t* = −11.8, df = 19, *p* < .001) and the mean *N*_*m*_ was significantly greater for local than regional pairs (Figure 2a; *t* = 5.1, df = 12, *p* < .001). For *An. punctulatus* in East Sepik, mean pairwise *F*_*ST*_ was significantly lower before (2008) the LLIN program was implemented than after (2010) it was implemented (Figure 2b; *t* = −4.7, df = 8, *p* = .002). Correspondingly, the mean pairwise *N*_*m*_ was significantly higher before than after the LLIN program was implemented (Figure 2b; *t* = 3.9, df = 6, *p* = .008).

### 3.3 Genetic clusters

**FIGURE 3:**
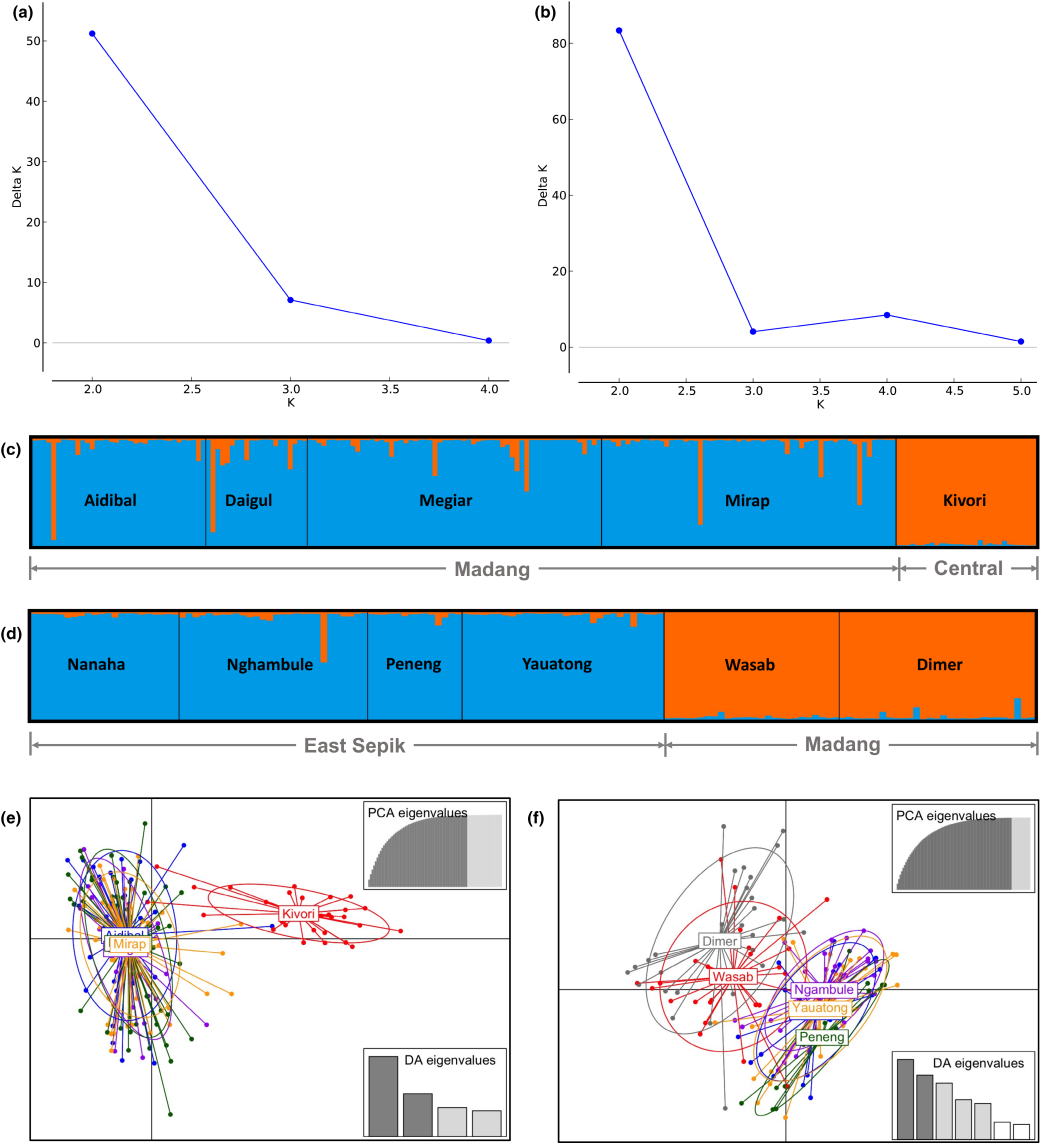
Plots of population structure from analysis of samples of *Anopheles farauti* from five villages in two provinces in 2017 (excluding Matukar as samples were unavailable for the year 2017) and of *Anopheles punctulatus* from six villages in two provinces in 2010 or 2012. (a) Bayesian estimates of Δ*K* for different values of *K* for *An. farauti*. (b) Estimates of Δ*K* for different values of *K* for *An. punctulatus*. (c) Bayesian structure plot for *K* = 2 for *An. farauti*. (d) Structure plot for *K* = 2 for *An. punctulatus*. In the Bayesian plots, columns represent individual mosquitoes and colors represent populations. (e) DAPC cluster plots for *An. farauti*. (f) DAPC cluster plots for *An. punctulatus*. In the DAPC plots, points represent individuals and colors represent the village they were collected. Ellipses represent clusters and lines connect individual mosquitoes to the center of gravity of the ellipse.

Bayesian and DAPC cluster analyses were performed on two of the three groups of mosquitoes that were analyzed with AMOVA. These were *An. punctulatus* collected in East Sepik and Madang in 2010 or 2012, and *An. farauti* in Madang and Central province in 2017. These two groups of mosquitoes show evidence of regional or subregional population structure based on the AMOVA test, which warranted further evaluation with the cluster analysis methods. The results of Evanno method show that for both *An. farauti* (Figure 3a) and *An. punctulatus* (Figure 3b), Δ*K* was highest for *K* = 2. This means that for both vector species, the mosquitoes clustered into two distinct populations. The Bayesian structure plots for both vector species are shown for *K* = 2 (Figure 3) as well as for higher values of *K* (Figure S4). For *An. farauti*, the mosquitoes from Madang localities clustered together to form one population which was genetically distinct from Kivori (Figure 3c). For *An. punctulatus*, the mosquitoes from East Sepik localities were clustered into one population and those from Madang clustered together into a separate population (Figure 3d). The result of DAPC was consistent with that of the Bayesian structure analysis. *An. farauti* formed two distinct clusters corresponding to the northern and southern plains (Figure 3e). Similarly, *An. punctulatus* formed two distinct clusters corresponding to the two subregions within the northern plain (Figure 3f). Although AMOVA detected post‐LLIN genetic differentiation of *An. punctulatus* within subregions (Table 2), this was not detected in the two cluster analyses.

### 3.4 Genetic diversity and bottleneck

Data of *An. farauti* from different localities within the Madang population were grouped into three groups according to the year of collection, combined with the population from Central province (one group) to form four groups of mosquitoes and estimates of genetic diversity were calculated (Table 3). Similarly, data of *An. punctulatus* from different localities within the East Sepik population were grouped into two groups according to the year of collection, combined with the Madang population (one group) to form three groups and genetic diversity estimates were calculated (Table 4). Among the four samples of *An. farauti*, the mean values (averaged across loci) of *N*_*A*_ ranged from 5.0–8.4, *A*_*R*_ was 4.7–5.6, *H*_*O*_ was 0.49–0.54, *H*_*E*_ was 0.52–0.56 and *uH*_*E*_ was 0.52–0.57 (Table 3). Among the three samples of *An. punctulatus*, the values of *N*_*A*_ ranged from 8.1–9.4, *A*_*R*_ was 7.7–7.9, *H*_*O*_ was 0.58–0.6, *H*_*E*_ was 0.65–0.68 and *uH*_*E*_ was 0.66–0.68 (Table 4). Data showing a decline in these genetic diversity estimates over time may indicate that a population has undergone or is undergoing a bottleneck event. However, this approach may not be robust as it is insensitive to mild genetic bottleneck. As such, it must be used alongside other formal, robust tests such as the LCH test. In this study, none of the genetic diversity estimates for both species (Tables 3 and 4) show a pattern of consistent declining values over time (no statistical test was necessary). *P* values for the LCH tests, which identify significant departure from mutation‐drift equilibrium (see Section 2: Materials and Methods) are presented for *An. farauti* in Table 3 and *An. punctulatus* in Table 4. Consistent with the results of genetic diversity estimates, genetic bottleneck was not observed in any of the *An. farauti* populations as LCH tests were not significant (Table 3). There was also no evidence of genetic bottleneck in the Madang population of *An. punctulatus* (Table 4). However, for *An. punctulatus* in East Sepik, genetic bottleneck was observed (LCH test, *p* = .007) after the LLIN campaign (2010) but not before (2008) the event (Table 4).

**TABLE 3:** Measures of genetic diversity including *p* values for the bottleneck tests for *Anopheles farauti*.

Population	Year	*n*	*N*_*A*_	*A*_*R*_	*H*_*O*_	*H*_*E*_	*uH*_*E*_	*p*
Madang	2010	74	7.5 (0.87)	5.6 (0.55)	0.54 (0.06)	0.54 (0.06)	0.54 (0.06)	.862
Madang	2012	65	6.7 (0.68)	5.4 (0.54)	0.53 (0.06)	0.52 (0.07)	0.52 (0.07)	.839
Madang	2017	179	8.4 (0.94)	5.5 (0.64)	0.52 (0.07)	0.54 (0.07)	0.54 (0.07)	.722
Central	2017	29	5.0 (0.60)	4.7 (0.53)	0.49 (0.06)	0.56 (0.06)	0.57 (0.06)	.161

*Note*: Values outside parenthesis are the means (averaged across loci) and inside parenthesis are the standard errors.

Abbreviations: *A*_*R*_, allelic richness; *H*_*E*_, expected heterozygosity; *H*_*O*_, observed heterozygosity; *n*, number of mosquitoes in the sample (i.e., sample size); *N*_*A*_, number of observed alleles; *p*, *p* value of the LCH tests; *uH*_*E*_, unbiased expected heterozygosity.

**TABLE 4:** Measures of genetic diversity including *p* values for the bottleneck tests for *Anopheles punctulatus*.

Population	Year	*n*	*N*_*A*_	*A*_*R*_	*H*_*O*_	*H*_*E*_	*uH*_*E*_	*p*
East Sepik	2008	120	9.4 (0.83)	7.9 (0.74)	0.58 (0.06)	0.65 (0.06)	0.66 (0.06)	.161
East Sepik	2010	94	8.6 (0.92)	7.8 (0.80)	0.58 (0.06)	0.68 (0.06)	0.68 (0.06)	**.007**
Madang	2012	55	8.1 (0.74)	7.7 (0.69)	0.60 (0.07)	0.67 (0.06)	0.68 (0.06)	.161

*Note*: Values outside parenthesis are the means (averaged across loci) and inside parenthesis are the standard errors.

Abbreviations: *A*_*R*_, allelic richness; *H*_*E*_, expected heterozygosity; *H*_*O*_, observed heterozygosity; *n*, number of mosquitoes in the sample (i.e., sample size); *N*_*A*_, number of observed alleles; *p*, *p* value of the LCH tests (bold font indicate significant results); *uH*_*E*_, unbiased expected heterozygosity.

## 4 DISCUSSION

The analysis of population genetic structure was performed at three spatial levels: regional (northern and southern plains), subregional (provinces within a region), and local (villages within a province). Analysis of population structure at the regional level could only be performed with *An. farauti* as *An. punctulatus* samples were all from a single region (northern plain). The results of *F*_*ST*_, AMOVA, and the two cluster analyses identified two distinct panmictic populations of *An. farauti* corresponding to the two regional plains, indicating restricted gene flow across the mountain ranges dividing the two plains. The population structure described here was based on samples of *An. farauti* collected after the LLIN campaign had been implemented. Pre‐LLIN samples of *An. farauti* were not available for comparison with the post‐LLIN results. However, post‐LLIN population structure observed here was consistent with a pre‐LLIN study by Ambrose et al. (2012) based on cytochrome oxidase I haplotype networks and pairwise *F*_*ST*_ analysis which observed genetic clustering between the northern and southern plains because of the mountain ranges (Ambrose et al., 2012).

Within the northern plain of PNG, there may be areas with environments (e.g., mountain ranges) unfavorable for high vector abundance but enough mosquitoes to maintain gene flow and population continuity within the region. Introduction of a vector intervention such as the LLIN into such areas can suppress vector abundance to the extent that gene flow is interrupted, causing genetic differentiation that can result in the formation of subpopulations. Similarly, populations whose range extends over long geographic distances are prone to fragmentation into subpopulations. Given the negative impact of LLIN intervention on vector abundance in the northern plain (Hetzel et al., 2016; Reimer et al., 2013, 2016), it is possible to observe a large northern metapopulation to fragment into smaller subpopulations. The results of *F*_*ST*_, AMOVA, and cluster analyses show that *An. punctulatus* in the northern plain form two distinct populations corresponding to the two subregions within it. That is, *An. punctulatus* from different localities in East Sepik constitute a single population whereas those from different localities in Madang constitute a single population separate from the East Sepik one. This result was based on data of mosquitoes that were collected after the LLIN campaign had been implemented. Pre‐LLIN samples of *An. punctulatus* from Madang were unavailable to enable comparison with the post‐LLIN results. However, the result of microsatellite analysis of pre‐LLIN samples of *An. punctulatus* in both provinces by Seah et al. (2013) contradicts that of the current study and shows that *An. punctulatus* in these provinces were genetically homogeneous, constituting a single panmictic population (Seah et al., 2013). Therefore, the results of the current (post‐LLIN) and published (pre‐LLIN) studies suggest that consistent with the predicted outcome the reduction in vector abundance by LLIN may have caused the metapopulation of *An. punctulatus* in the northern plain to fragment into two subpopulations. For *An. farauti*, pre‐LLIN results of Ambrose et al. (2012) show genetic homogeneity of *An. farauti* samples from provinces within the northern plain. Unfortunately, samples from East Sepik were unavailable to make an inference about potential post‐LLIN population fragmentation between subregions in the northern plain in the current study.

For both vector species, neither Bayesian structure nor DAPC analyses detected local (fine scale) genetic clustering. However, for *An. punctulatus* in East Sepik, the results of AMOVA and mean pairwise *F*_*ST*_ show local genetic differentiation after the LLIN campaign was implemented but not before the event. In contrast, AMOVA and pairwise *F*_*ST*_ analyses did not show local genetic differentiation of *An. farauti*. These results indicate that LLINs had a mild effect on the population structure of *An. punctulatus* at a fine spatial scale, but did not have this effect on *An. farauti*.

Reduction in vector abundance can also affect the effective population size of the vectors, and both vector species experienced a reduction in their abundance after the LLINs were distributed (Hetzel et al., 2016; Reimer et al., 2013, 2016). However, in the current study, LCH tests did not detect post‐LLIN genetic bottlenecks in any populations of *An. farauti* or the Madang population of *An. punctulatus*. For the East Sepik population of *An. punctulatus*, a genetic bottleneck was detected. These results indicate a differential response of the malaria vectors to the LLIN intervention. The differential response of the two vector species to LLINs can be caused by one or more factors that are unknown to us. One way to explain this phenomenon is in terms of variation in their host selection. LLIN is an indoor‐focused vector intervention method which affects anthropophagic vectors (those that prefer human blood) more than opportunistic ones (those that feed on any available host). Although *An. farauti* and *An. punctulatus* are generally opportunistic in their selection of blood‐meal hosts, allowing them to support their abundance by feeding on domestic animal hosts (dogs and pigs) when access to human hosts is limited by the bed nets (Keven et al., 2017), *An. farauti* is relatively more flexible than *An. punctulatus* and easily resorts to the animal hosts when human hosts are not readily accessible (J. B. Keven, M. Katusele, R. Vinit, D. Rodríguez‐Rodríguez, M. W. Hetzel, L. J. Robinson, M. Laman, S. Karl and E. D. Walker, unpublished data). This slight variation in their host selection may explain the variation in genetic bottleneck observed between *An. punctulatus* and *An. farauti* in the current study. It is unclear why the East Sepik population of *An. punctulatus* experienced a genetic bottleneck but not the Madang population. Factors such as heterogeneity in insecticide resistance and the rates of bed net usage in different provinces are potential explanations. However, the natural populations of *Anopheles* mosquitoes in PNG have remained fully susceptible to the bed nets (Katusele et al., 2014; Keven et al., 2010; Koimbu et al., 2018), and published data on bed net usage by province during the time the mosquito samples in this study were collected (2010–2012) are not available to support this speculation. Another possible explanation for the variation in genetic bottleneck between the two species is the variation in their genetic diversity. The genetic structure of *An. farauti* appears to be more stable as the observed and expected heterozygosity were nearly equal. In contrast, the genetic structure of *An. punctulatus* appears to be unstable, as some factors, possibly inbreeding or Wahlund effect appear to increase the homozygosity in this vector species. Although the abundance of both vector species was reduced by the LLINs, the one with unstable populations (*An. punctulatus*) is more likely to experience a genetic bottleneck event.

This study has three caveats. First, the mosquito specimens analyzed here did not represent samples from a longitudinal collection from 2008 to 2017. Therefore, environmental factors associated with seasonality (wet/dry periods) could potentially affect population size and could be misinterpreted here as attributed to the LLIN program. However, unlike sub‐Saharan Africa, seasonality is not likely to have a profound effect on the abundance of PNG vectors, at least not to the extent that may affect the effective population size (Burkot et al., 1988, 1989; Charlwood et al., 1986; Hetzel et al., 2016; Hii et al., 1997, 2001; Keven et al., 2017; Keven, Katusele, et al., 2019; Reimer et al., 2016). Second, there is evidence that LLINs distributed in PNG that were manufactured after 2013 were substandard and had low efficacy against known susceptible *Anopheles* mosquitoes in PNG (Vinit et al., 2020). This can cause re‐expansion of the vector population abundance (which has been observed elsewhere (Keven et al., 2022)), thereby reversing any bottleneck effect that may have happened early in the vector control effort. Third, the assertion that opportunistic host selection in the two vector species sustains their abundance in the presence of LLIN intervention contradicts the premise of this study which is that vector abundance was reduced significantly following the vector intervention. To clarify this potential misunderstanding, while the bed nets had a significant impact on the census size of the mosquito populations, its impact on the population's genetic parameters was limited by the host selection factor. That is, the ability of the vectors to resort to alternative hosts kept the bed nets from suppressing the mosquito numbers passed the threshold below which the effective population size can be affected.

To conclude, this study was set out to test whether the reduction in the abundance of the malaria vectors, *An. farauti* and *An. punctulatus*, as a consequence of the LLIN‐based vector intervention in PNG in 2009 affected their effective population size and reshaped their population genetic structure. The results show evidence of genetic bottleneck, an indicator of reduced effective population size in *An. punctulatus* but not *An. farauti*. Similarly, analysis of population structure shows fragmentation of metapopulation of *An. punctulatus* into isolated subpopulations but not *An. farauti*, although further analysis is needed to confirm this observation for the latter vector species. These findings show a differential response of the vectors to the malaria control program in PNG, indicating that the LLINs may not be effective against some vector species. Future programs that evaluate the impact of malaria vector interventions should incorporate population genetic studies to determine the severity of the intervention on the vectors.

## AUTHOR CONTRIBUTIONS

**John B. Keven:** Conceptualization (lead); data curation (lead); formal analysis (lead); investigation (lead); methodology (lead); supervision (lead); writing – original draft (lead); writing – review and editing (lead). **Rebecca Vinit:** Data curation (supporting); formal analysis (supporting); investigation (equal); methodology (supporting); writing – review and editing (supporting). **Michelle Katusele:** Conceptualization (supporting); data curation (supporting); formal analysis (supporting); investigation (equal); methodology (equal); writing – review and editing (supporting). **Lisa J. Reimer:** Data curation (supporting); formal analysis (supporting); funding acquisition (supporting); investigation (equal); methodology (supporting); project administration (supporting); writing – review and editing (equal). **Peter A. Zimmerman:** Conceptualization (supporting); data curation (supporting); funding acquisition (lead); investigation (equal); methodology (supporting); project administration (supporting); supervision (supporting); writing – review and editing (supporting). **Stephan Karl:** Conceptualization (equal); data curation (equal); formal analysis (supporting); funding acquisition (supporting); investigation (equal); methodology (supporting); project administration (lead); supervision (equal); writing – original draft (supporting); writing – review and editing (equal). **Edward D. Walker:** Conceptualization (equal); data curation (equal); formal analysis (equal); funding acquisition (lead); investigation (equal); methodology (equal); project administration (equal); supervision (equal); validation (equal); writing – original draft (equal); writing – review and editing (equal).

## CONFLICT OF INTEREST STATEMENT

The authors declare that they have no competing interests.

## Supporting information


Figure S1–S4.

Table S1–S5.

Data S1.

Data S2.

## Data Availability

Data supporting the conclusions of this article are included within this article and its supplementary files.
