# Supplementary material for: Genetic differentiation and bottleneck effects in the malaria vectors Anopheles farauti and Anopheles punctulatus after an LLIN‐based vector control program in Papua New Guinea
Source: Ecol Evol. 2024 Feb 15;14(2):e10917. doi: 10.1002/ece3.10917 (PMC10869881; doi:10.1002/ece3.10917)
Supplement: Supplementary file 1 — Figure S1–S4. [file ECE3-14-e10917-s004.docx]

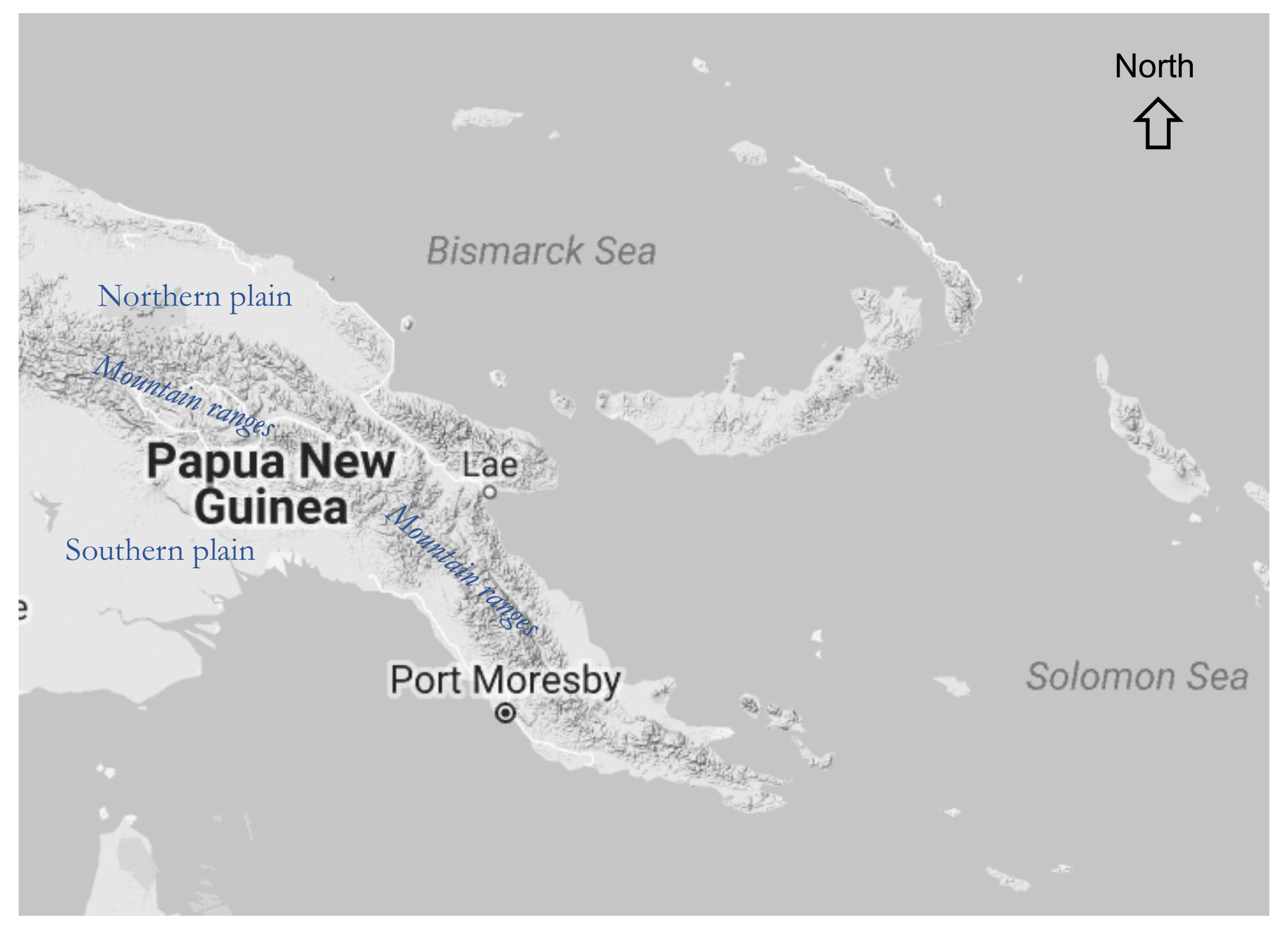


Fig. S1. Map of Papua New Guinea showing the connected mountain ranges separating the northern and southern lowland plains.


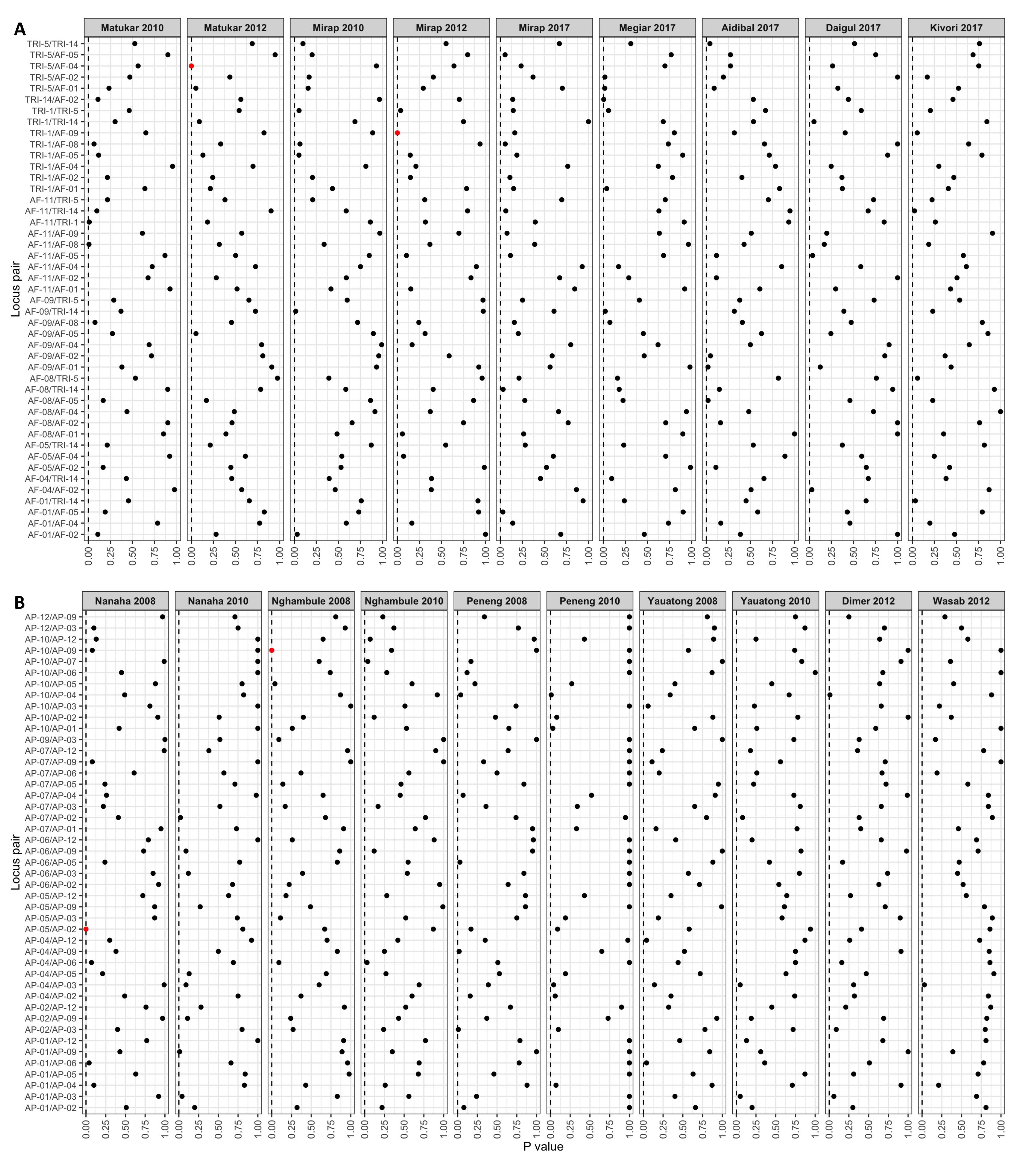


Fig. S2. P values of linkage disequilibrium (LD) tests for pairs of microsatellite loci in each sample of *An. farauti* (s.s.) (panel A) and *An. punctulatus* (s.s.) (panel B). Broken vertical lines represent the Bonferroni-corrected alpha. P values represented by black dots are pairs of loci that were statistically found to be unliked (i.e., fall to the right of the broken vertical line) whereas those represented by red dots are pairs that were found to be genetically linked (i.e., fall to the left of the vertical line).


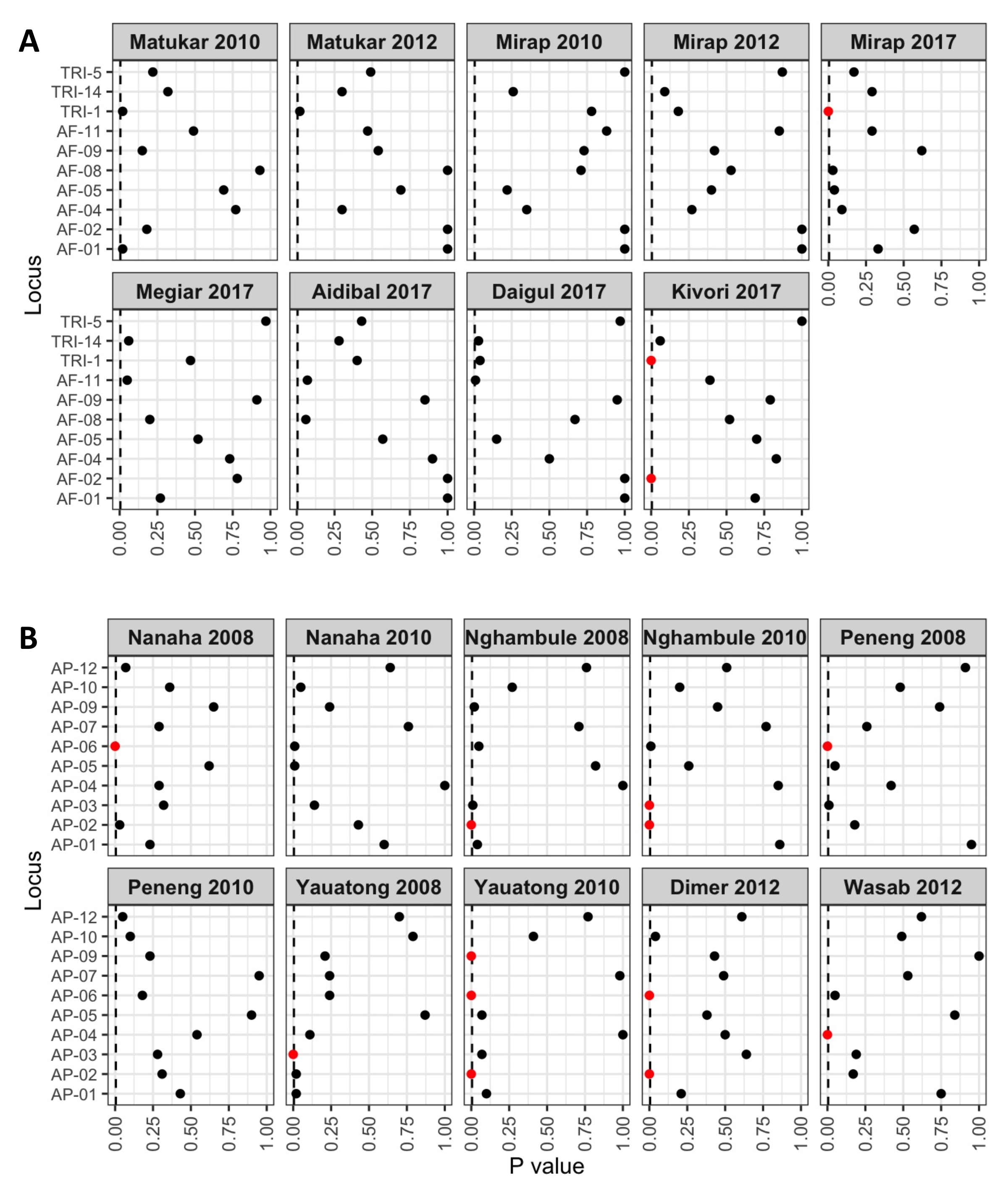


Fig. S3. P values of Hardy-Weinberg equilibrium (HWE) tests for each microsatellite locus in each sample of *An. farauti* (s.s.) (panel A) and *An. punctulatus* (s.s.) (panel B). Broken vertical lines represent the Bonferroni-corrected alpha. P values represented by black dots are loci that were in HWE (i.e., fall to the right of the broken vertical line) whereas those represented by red dots are loci that deviated from HWE (i.e., fall to the left of the vertical line).


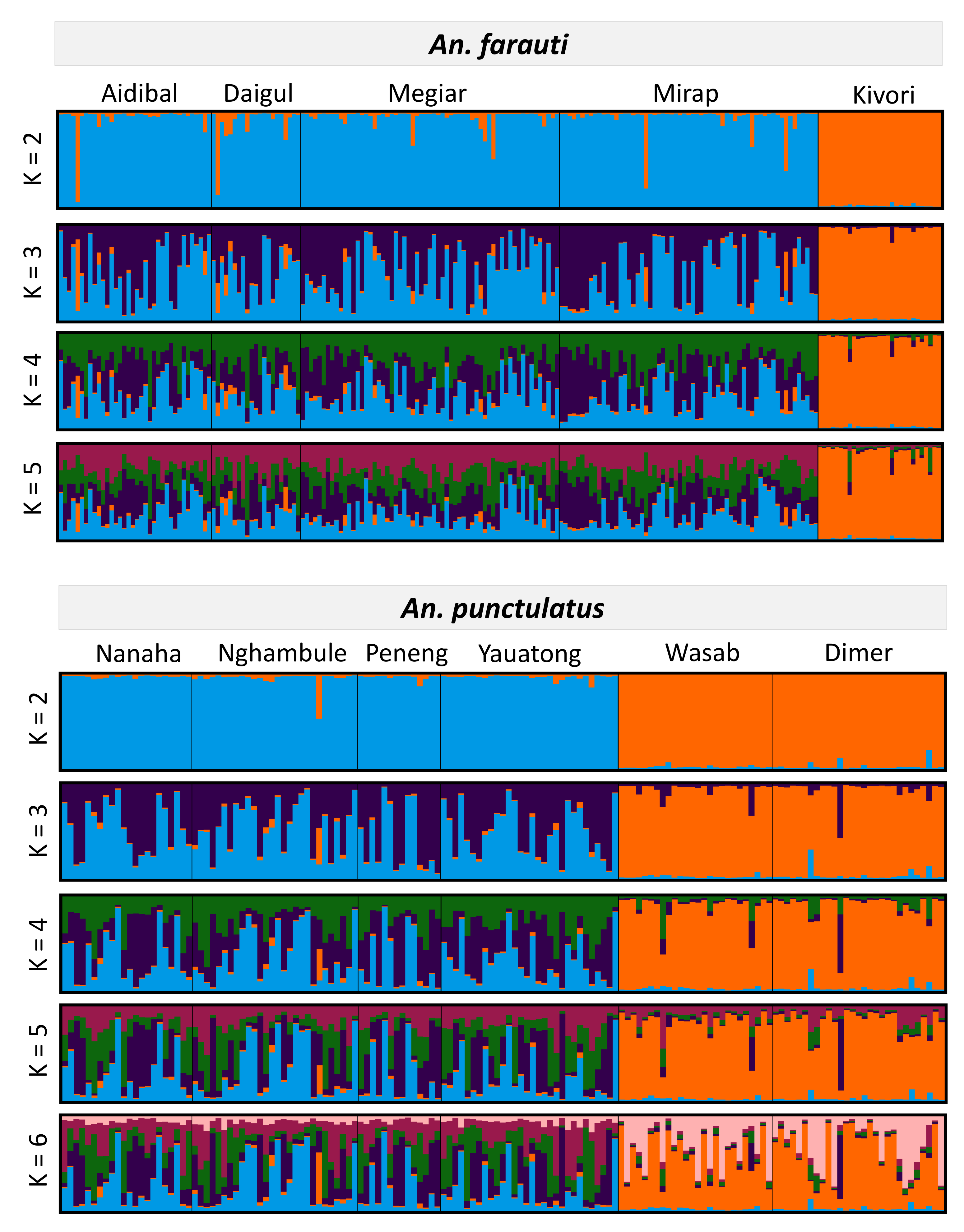


Fig. S4. Structure plots for all values of *K* for *An. farauti* (*K* = 2 to 5) and *An. punctulatus* (*K* = 2 to 6).
