# Supplementary material for: Genetic differentiation and bottleneck effects in the malaria vectors Anopheles farauti and Anopheles punctulatus after an LLIN‐based vector control program in Papua New Guinea
Source: Ecol Evol. 2024 Feb 15;14(2):e10917. doi: 10.1002/ece3.10917 (PMC10869881; doi:10.1002/ece3.10917)
Supplement: Supplementary file 2 — Table S1–S5. [file ECE3-14-e10917-s002.docx]

Table S1. Microsatellite repeat motif, primer sequence and allele range for *An. farauti*.

| Name | Repeat motif | Primer sequence (5’–3’) | Allele range (in base pairs) |
| --- | --- | --- | --- |
| AF-11 | (GAAA)_n_ | **CTCACCTCCCACTCCACAAA**AATACCACGGCGAACGAA  **GTTTCTT**GCGTGCCTTTATGCCTTTTA | 172–198 |
| TRI-1* | (TGC)_n_ | **AAACCTCTCTCCACACCCAAA**ATGTGGTCGGCTGTTTATGG  **GTTTCTT**GCGTAAACACATGTGCGATC | 168–195 |
| AF-09 | (TCCA)_n_ | **CTCCAACTCACCTCCAACAAA**GATGATTCGATTCGGCAAA  **GTTTCTT**CGCTCTGAGACATCACTTGAA | 183–248 |
| AF-08 | (TCCA)_n_ | **AACTCCACCACTCCCACAAA**CCTGGGGAAGAGGAATCAA  **GTTTCTT**GCAAGGCGTCACTACCAAA | 260–297 |
| TRI-5* | (CAG)_n_ | **AAACCTCTCTCCACACCCAAA**TTTAGCTGCAACACGACGAC  **GTTTCTT**CGCGAGAAAGAGGAAACCAC | 263–299 |
| AF-01 | (TGAA)_n_ | **CTCCAACTCACCTCCAACAAA**CCGATTTCTTCTTACGTCCTACAA  **GTTTCTT**TACGAACTGCACAACAGCAA | 263–300 |
| AF-05 | (GAT)_n_ | **AACTCCACCACTCCCACAAA**CTCAAGCTGAGTCCGTCCAA  **GTTTCTT**ACAATCTATGCCGTCAGCAA | 345–386 |
| AF-04 | (AACA)_n_ | **CTCACCTCCCACTCCACAAA**GATGCGTACTTCGAGTGCAA  **GTTTCTT**GTGCTGTGGGAAGAAAGTGAA | 287–398 |
| TRI-14* | (GAC)_n_ | **AAACCTCTCTCCACACCCAAA**TTGTGCGGGATGAAATAGCG  **GTTTCTT**AGGCAAAGAGTCGGTCAGAG | 377–418 |
| AF-02 | (TGGA)_n_ | **CTCCAACTCACCTCCAACAAA**GTTAAATCGATCGGCGAAA  **GTTTCTT**CGGTGAATGGATGAAGACAA | 377–402 |

Markers with an asterisk (*) were adopted elsewhere (Ambrose et al. 2014, *Int. J. Parasitol*. 44; 225–233). Bold font segment of the reverse primers is the pig tail sequence GTTTCTT and that of the forward primers is the sequence complementary to a universal primer. The forward primer of markers AF-09, AF-01 and AF-02 had the tag sequence complementary to universal primer 1 (6FAM-CTCCAACTCACCTCCAACAAA), markers TRI-1, TRI-5 and TRI-14 had the sequence complementary to universal primer 2 (PET-AAACCTCTCTCCACACCCAAA), markers AF-11 and AF-04 had the sequence complementary to universal primer 3 (NED-CTCACCTCCCACTCCACAAA), and markers AF-08 and AF-05 had the sequence complementary to universal primer 4 (VIC- AACTCCACCACTCCCACAAA).

Table S2. Microsatellite repeat motif, primer sequence and allele range for *An. punctulatus*.

| Name | Repeat motif | Primer sequences (5’–3’) | Allele range (in base pairs) |
| --- | --- | --- | --- |
| AP-10 | (AGCC)_n_ | **AACTCCACCACTCCCACAAA**TGGGTAGGAAAATGTGTGGAA  **GTTTCTT**CAATCCACAGGAAGCATACAAA | 183–241 |
| AP-07 | (CAAA)_n_ | **CTCACCTCCCACTCCACAAA**CGGTATTGGTGTGGCTTCAA  **GTTTCTT**CGGTTGGGTCAAGTGGAA | 196–237 |
| AP-01 | (CAAG)_n_ | **CTCCAACTCACCTCCAACAAA**AAGGCAGACGAAAGAAGCAA  **GTTTCTT**TCTGTATGTTTGCTTGACCTTACAA | 175–223 |
| AP-04 | (AACG)_n_ | **AACTCCACCACTCCCACAAA**CCGATACGGTTGACGCTAA  **GTTTCTT**GTGATCGCCAGCATCAGAA | 270–312 |
| AP-06 | (CCAT)_n_ | **CTCACCTCCCACTCCACAAA**AGGTCGCGACGATCAAGAA  **GTTTCTT**GTCTTGACGGATGGATGGAA | 282–329 |
| AP-05 | (AACG)_n_ | **AAACCTCTCTCCACACCCAAA**TCTTGCCAGCAGGAAGAAAA  **GTTTCTT**GGAATGCAAGTAAGCGTACGAA | 277–296 |
| AP-02 | (TCAT)_n_ | **CTCCAACTCACCTCCAACAAA**GAACCGCTTCTTTAACCACAA  **GTTTCTT**AGGCAGAAAGGCTGAAGGTAA | 281–306 |
| AP-12 | (CAAA)_n_ | **AACTCCACCACTCCCACAAA**GGGGTTTGGTTGGGTTAGAA  **GTTTCTT**AGCTCGTATGCGAAGGACAA | 360–397 |
| AP-09 | (CCAG)_n_ | **CTCACCTCCCACTCCACAAA**GGCGTGGATGAACAAAGAA  GTTTCTTTCTGCACCGTCTGGAACAA | 375–414 |
| AP-03 | (CCAT)_n_ | **CTCCAACTCACCTCCAACAAA**GCGGAATGTGCAATCAATAA  **GTTTCTT**GGCAGTTTCCAAACACGAA | 369–408 |

Bold font segment of the reverse primers is the pig tail sequence GTTTCTT and that of the forward primers is the sequence complementary to a universal primer. The forward primer of markers AP-01, AP-02 and AP-03 had the tag sequence complementary to universal primer 1, marker AP-05 had the sequence complementary to universal primer 2, markers AP-06, AP-07 and AP-09 had the sequence complementary to universal primer 3, and markers AP-04, AP-10 and AP-12 had the sequence complementary to universal primer 4 (see Table S1 for the universal primers).

Table S3. Frequency of null alleles for each microsatellite marker as well as average and standard error (SE) estimated across loci for nine samples of *An. farauti* (*s.s.*).

| Marker | Aidibal 2017 | Daigul 2017 | Kivori 2017 | Matukar 2010 | Matukar 2012 | Megiar 2017 | Mirap 2010 | Mirap 2012 | Mirap 2017 |
| --- | --- | --- | --- | --- | --- | --- | --- | --- | --- |
| AF-11 | 0.09 | 0.10 | 0.03 | 0.01 | 0.00 | 0.04 | 0.00 | 0.00 | 0.00 |
| TRI-1 | 0.03 | 0.07 | 0.20 | 0.07 | 0.00 | 0.00 | 0.02 | 0.04 | 0.04 |
| AF-09 | 0.04 | 0.00 | 0.00 | 0.00 | 0.00 | 0.02 | 0.00 | 0.00 | 0.02 |
| AF-08 | 0.10 | 0.03 | 0.00 | 0.00 | 0.00 | 0.00 | 0.00 | 0.04 | 0.06 |
| TRI-5 | 0.00 | 0.00 | 0.00 | 0.03 | 0.00 | 0.00 | 0.00 | 0.00 | 0.07 |
| AF-01 | 0.00 | 0.00 | 0.01 | 0.06 | 0.00 | 0.04 | 0.00 | 0.00 | 0.04 |
| AF-05 | 0.00 | 0.06 | 0.01 | 0.00 | 0.02 | 0.05 | 0.00 | 0.00 | 0.07 |
| AF-04 | 0.00 | 0.00 | 0.00 | 0.00 | 0.03 | 0.00 | 0.03 | 0.00 | 0.03 |
| TRI-14 | 0.00 | 0.05 | 0.03 | 0.05 | 0.05 | 0.00 | 0.04 | 0.00 | 0.01 |
| AF-02 | 0.00 | 0.00 | 0.20 | 0.00 | 0.00 | 0.00 | 0.00 | 0.00 | 0.02 |
| Mean | 0.03 | 0.03 | 0.05 | 0.02 | 0.01 | 0.02 | 0.01 | 0.01 | 0.04 |
| SE | 0.01 | 0.01 | 0.03 | 0.01 | 0.01 | 0.01 | 0.00 | 0.01 | 0.01 |

Table S4. Frequency of null alleles for each microsatellite marker as well as average and standard error (SE) estimated across loci for 10 samples of *An. punctulatus* (*s.s.*).

| Marker | Dimer 2012 | Nanaha 2008 | Nanaha 2010 | Nghambule 2008 | Nghambule 2010 | Peneng 2008 | Peneng 2010 | Wasab 2012 | Yauatong 2008 | Yauatong 2010 |
| --- | --- | --- | --- | --- | --- | --- | --- | --- | --- | --- |
| AP-10 | 0.10 | 0.00 | 0.04 | 0.03 | 0.04 | 0.04 | 0.09 | 0.03 | 0.03 | 0.04 |
| AP-07 | 0.00 | 0.00 | 0.00 | 0.00 | 0.00 | 0.00 | 0.00 | 0.00 | 0.00 | 0.00 |
| AP-01 | 0.00 | 0.00 | 0.00 | 0.00 | 0.00 | 0.00 | 0.08 | 0.06 | 0.10 | 0.06 |
| AP-04 | 0.00 | 0.00 | 0.00 | 0.00 | 0.00 | 0.05 | 0.04 | 0.12 | 0.00 | 0.00 |
| AP-06 | 0.24 | 0.14 | 0.17 | 0.11 | 0.13 | 0.14 | 0.08 | 0.09 | 0.05 | 0.21 |
| AP-05 | 0.06 | 0.00 | 0.13 | 0.00 | 0.00 | 0.00 | 0.00 | 0.00 | 0.00 | 0.09 |
| AP-02 | 0.19 | 0.07 | 0.05 | 0.20 | 0.16 | 0.07 | 0.05 | 0.10 | 0.13 | 0.19 |
| AP-12 | 0.00 | 0.05 | 0.03 | 0.00 | 0.02 | 0.00 | 0.00 | 0.00 | 0.04 | 0.00 |
| AP-09 | 0.04 | 0.00 | 0.00 | 0.14 | 0.03 | 0.00 | 0.06 | 0.00 | 0.07 | 0.16 |
| AP-03 | 0.02 | 0.08 | 0.00 | 0.12 | 0.21 | 0.15 | 0.04 | 0.07 | 0.19 | 0.11 |
| Mean | 0.06 | 0.03 | 0.04 | 0.06 | 0.06 | 0.04 | 0.04 | 0.05 | 0.06 | 0.09 |
| SE | 0.03 | 0.02 | 0.02 | 0.02 | 0.02 | 0.02 | 0.01 | 0.01 | 0.02 | 0.03 |

Table S5. Results for pairwise *F_ST_* and *N_m_*.

| Vector | Pairs | Type | Group | *F_ST_* | *N_m_* |
| --- | --- | --- | --- | --- | --- |
| *An. farauti* | Aidibal 2017/Daigul 2017 | Local | Post-LLIN | 0.008 | 32.583 |
| *An. farauti* | Aidibal 2017/Megiar 2017 | Local | Post-LLIN | 0.004 | 56.624 |
| *An. farauti* | Aidibal 2017/Mirap 2017 | Local | Post-LLIN | 0.005 | 54.301 |
| *An. farauti* | Daigul 2017/Megiar 2017 | Local | Post-LLIN | 0.008 | 32.032 |
| *An. farauti* | Daigul 2017/Mirap 2017 | Local | Post-LLIN | 0.008 | 32.873 |
| *An. farauti* | Megiar 2017/Mirap 2017 | Local | Post-LLIN | 0.002 | 102.922 |
| *An. farauti* | Aidibal 2017/Kivori 2017 | Regional | Post-LLIN | 0.066 | 3.515 |
| *An. farauti* | Daigul 2017/Kivori 2017 | Regional | Post-LLIN | 0.068 | 3.403 |
| *An. farauti* | Kivori 2017/Megiar 2017 | Regional | Post-LLIN | 0.060 | 3.910 |
| *An. farauti* | Kivori 2017/Mirap 2017 | Regional | Post-LLIN | 0.059 | 3.997 |
| *An. punctulatus* | Dimer 2012/Wasab 2012 | Local | Post-LLIN | 0.014 | 18.206 |
| *An. punctulatus* | Nanaha 2010/Nghambule 2010 | Local | Post-LLIN | 0.017 | 14.821 |
| *An. punctulatus* | Nanaha 2010/Peneng 2010 | Local | Post-LLIN | 0.018 | 13.837 |
| *An. punctulatus* | Nanaha 2010/Yauatong 2010 | Local | Post-LLIN | 0.011 | 22.156 |
| *An. punctulatus* | Nghambule 2010/Peneng 2010 | Local | Post-LLIN | 0.020 | 12.010 |
| *An. punctulatus* | Nghambule 2010/Yauatong 2010 | Local | Post-LLIN | 0.013 | 18.727 |
| *An. punctulatus* | Peneng 2010/Yauatong 2010 | Local | Post-LLIN | 0.012 | 20.584 |
| *An. punctulatus* | Nanaha 2008/Nghambule 2008 | Local | Pre-LLIN | 0.009 | 26.341 |
| *An. punctulatus* | Nanaha 2008/Peneng 2008 | Local | Pre-LLIN | 0.007 | 37.580 |
| *An. punctulatus* | Nanaha 2008/Yauatong 2008 | Local | Pre-LLIN | 0.008 | 30.180 |
| *An. punctulatus* | Nghambule 2008/Peneng 2008 | Local | Pre-LLIN | 0.009 | 27.414 |
| *An. punctulatus* | Nghambule 2008/Yauatong 2008 | Local | Pre-LLIN | 0.004 | 57.914 |
| *An. punctulatus* | Peneng 2008/Yauatong 2008 | Local | Pre-LLIN | 0.005 | 46.300 |
| *An. punctulatus* | Dimer 2012/Nanaha 2010 | Regional | Post-LLIN | 0.032 | 7.586 |
| *An. punctulatus* | Dimer 2012/Nghambule 2010 | Regional | Post-LLIN | 0.031 | 7.779 |
| *An. punctulatus* | Dimer 2012/Peneng 2010 | Regional | Post-LLIN | 0.037 | 6.457 |
| *An. punctulatus* | Dimer 2012/Yauatong 2010 | Regional | Post-LLIN | 0.031 | 7.837 |
| *An. punctulatus* | Nanaha 2010/Wasab 2012 | Regional | Post-LLIN | 0.033 | 7.438 |
| *An. punctulatus* | Nghambule 2010/Wasab 2012 | Regional | Post-LLIN | 0.033 | 7.271 |
| *An. punctulatus* | Peneng 2010/Wasab 2012 | Regional | Post-LLIN | 0.037 | 6.589 |
| *An. punctulatus* | Wasab 2012/Yauatong 2010 | Regional | Post-LLIN | 0.027 | 9.083 |
